# Exploring the prognostic function of TMB-related prognostic signature in patients with colon cancer

**DOI:** 10.1186/s12920-023-01555-2

**Published:** 2023-05-26

**Authors:** Yan Zhao, Xiaolong Liang, Xudong Duan, Chengli Zhang

**Affiliations:** 1 Department of Nuclear Medicine, Zigong First People’s Hospital, Zigong, 643000 Sichuan PR China; 2 Department of Gastrointestinal Surgery, The First Affiliated Hospital of Chongqing Medical University, Chongqing, 400010 PR China; 3 Oncology Department, Zigong First People’s Hospital, Zigong, 643000 Sichuan PR China

**Keywords:** Tumor mutation burden, Prognosis, Risk signature, Colon cancer

## Abstract

Tumor mutation burden (TMB) level is identified as a useful predictor in multiple tumors including colon adenocarcinoma (COAD). However, the function of TMB related genes has not been explored previously. In this study, we obtained patients’ expression and clinical data from The Cancer Genome Atlas (TCGA) and the National Center for Biotechnology Information (NCBI). TMB genes were screened and subjected to differential expression analysis. Univariate Cox and LASSO analyses were utilized to construct the prognostic signature. The efficiency of the signature was tested by using a receiver operating characteristic (ROC) curve. A nomogram was further plotted to assess the overall survival (OS) time of patients with COAD. In addition, we compared the predictive performance of our signature with other four published signatures. Functional analyses indicated that patients in the low-risk group have obviously different enrichment of tumor related pathways and tumor infiltrating immune cells from that of high-risk patients. Our findings suggested that the ten genes’ prognostic signature could exert undeniable prognostic functions in patients with COAD, which might provide significant clues for the development of personalized management of these patients.

**Supplementary Information:**

The online version contains supplementary material available at 10.1186/s12920-023-01555-2.

## Introduction

Colon cancer (COAD) is the third most common malignant tumor and ranks the second leading tumor-related deaths worldwide [1]. With progress in the diagnosis and treatment of COAD, the morbidity and mortality of COAD have decreased. However, there were still many new cases and deaths each year [2]. According to the Cancer Statistics 2021, there were more than one million new COAD cases and 0.5 million deaths [1]. Considering the high morbidity and mortality of COAD, it is urgent to identify effective biomarkers to predict COAD patients’ prognosis.

COAD is a tumor with high heterogeneity, which resulted from a series of genetic mutations in tumor cells [3, 4]. Tumor mutation burden (TMB) is defined as the number of non-synonymous somatic coding errors per megabase in tumor cells [5]. Mutations of key genes could affect various biological functions of tumor cells [6]. In addition, mutations of tumor-related genes were reported to be associated with the prognosis of tumor patients [7, 8]. A recent study proved that mutation of FREM2 is related to the poor prognosis of COAD patients [9]. Apart from the prognosis prediction, TMB was also revealed to be correlated with the tumor microenvironment of COAD [10]. All these evidences indicated that TMB is closed correlated with COAD. However, the prognostic value of TMB-related genes in COAD has not been explored previously.

In this study, we constructed a TMB-related genes’ signature using the COAD dataset from The Cancer Genome Atlas (TCGA) database and validated the performance of signature in a GEO cohort. Then, GSEA analysis was used to identify the difference in the enrichment of pathways. We also explored the association between the signature and tumor immune microenvironment. Our findings could help to effectively predict the prognosis of COAD patients and might provide crucial clues for exploring new treatment strategies.

## Results

### Identification of differential TMB-related genes and construction of the risk signature

**Fig. 1:**
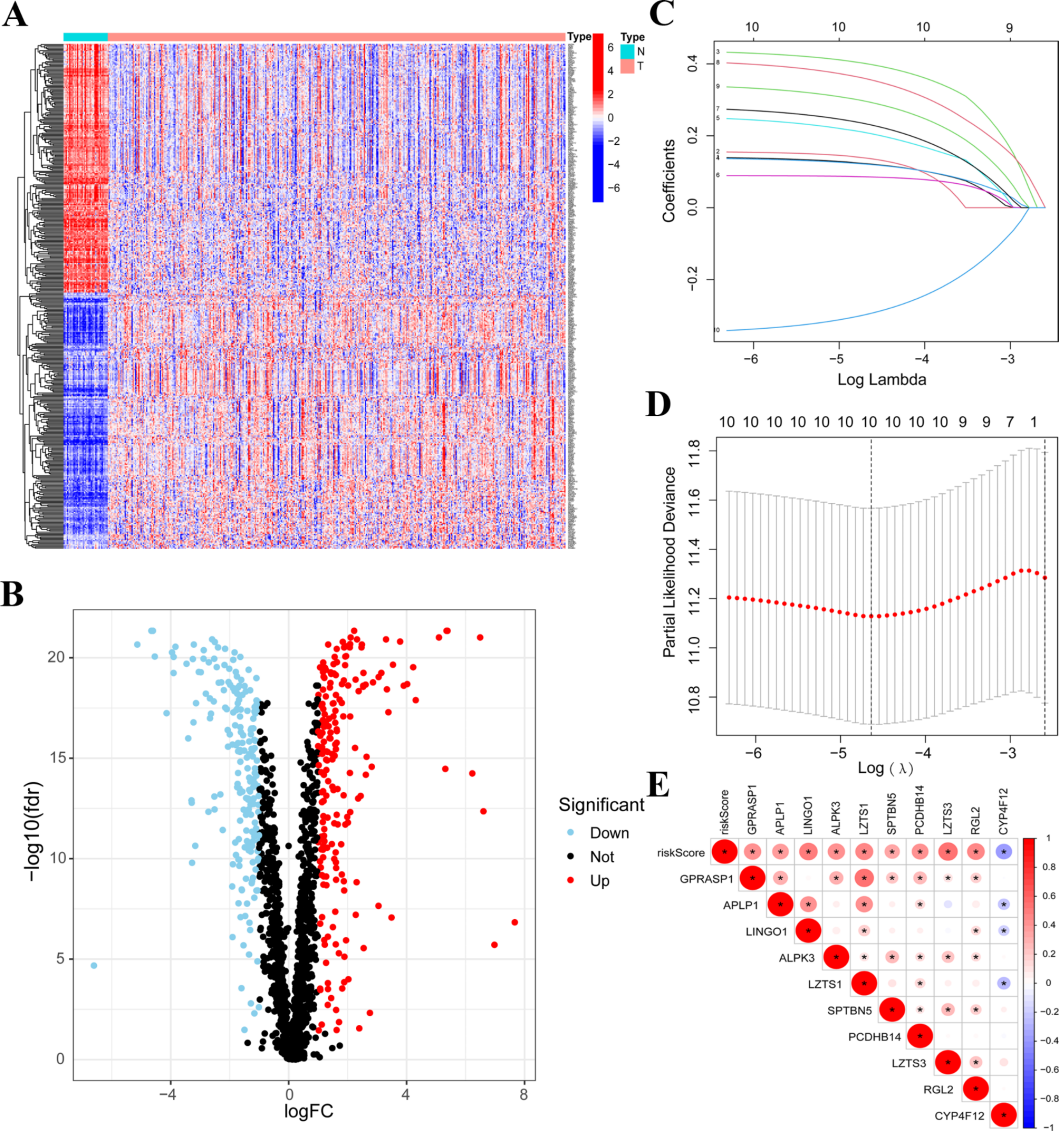
Establishment of the risk signature. (**A** and **B**) 410 differently expressed genes were visualized by using a heatmap and a volcano map. (**C** and **D**) LASSO regression analysis was utilized for the construction of risk signature. (**E**) The correlation between genes expression value and risk score value was visualized, *P < 0.05

To acquire the expression of Tumor mutation burden (TMB) related genes, the TMB data and RNA-seq data were download from The Cancer Genome Atlas (TCGA). The genes with mutation frequency more than in ten samples were extracted, which results in 2741 TMB-related genes (Supplementary Table 1). Subsequently, we screened differentially expressed genes among these 2741 TMB-related genes (*P* < 0.05, log_2_|FC|>1). 208 genes were identified to be upregulated and 202 genes were proved to be downregulated in tumor tissues compared with normal tissues (Fig. 1A and B). The details of these TBM-related genes were documented in Supplementary Table 2. After acquiring the differentially expressed TMB-related genes, we conducted univariate Cox analysis to identify genes with prognostic function. In total, ten TMB-related genes were obtained (Table 1).

**Table 1:** Univariable Cox results for TMB related genes in COAD.

Gene symbol	HR	(95%CI)	*P*-value
GPRASP1	1.627	1.177–2.250	0.003**
APLP1	2.016	1.348–3.468	0.001**
LINGO1	1.726	1.239–2.406	0.001**
ALPK3	1.391	1.112–1.739	0.004**
LZTS1	2.562	1.337–4.908	0.005**
SPTBN5 PCDHB14 LZTS3 RGL2 CYP4F12	1.775 1.424 1.608 1.814 0.639	1.190–2.648 1.088–1.863 1.224–2.113 1.203–2.737 0.469–0.872	0.005** 0.010* < 0.001*** 0.005** 0.005**

HR: hazard ratio; CI: confidence interval; *P < 0.05, **P < 0.01, ***P < 0.001

Then, ten TMB-related genes were subjected to LASSO Cox regression analysis to avoid co-linear influences, and regression coefficients value were calculated. Interestingly, we found that ten genes resulted from univariate Cox could achieve the best performance (Fig. 1C and D). The coefficients value of these ten genes were shown in Supplementary Table 3. The TMB information of these genes were shown in Supplementary Fig. 1A and 1B. We also explored the correlation among these genes, and detected the correlation between these genes with risk score. Results demonstrated that nine out of ten genes’ expression value were positively correlated with the risk score (Fig. 1E).

### Prognostic performance of the risk signature

To validate the prognostic function of the risk signature, TCGA cohort was first utilized as the training set. Then, patients in TCGA cohort were randomly divided in to validation set 1 and validation set 2 at a ratio of 1:1. We validated the performance of the risk signature in two inner validation sets. In addition, we also acquired data from the National Center for Biotechnology Information (GEO cohort). The data of GEO cohort was used as an external validation set.

**Fig. 2:**
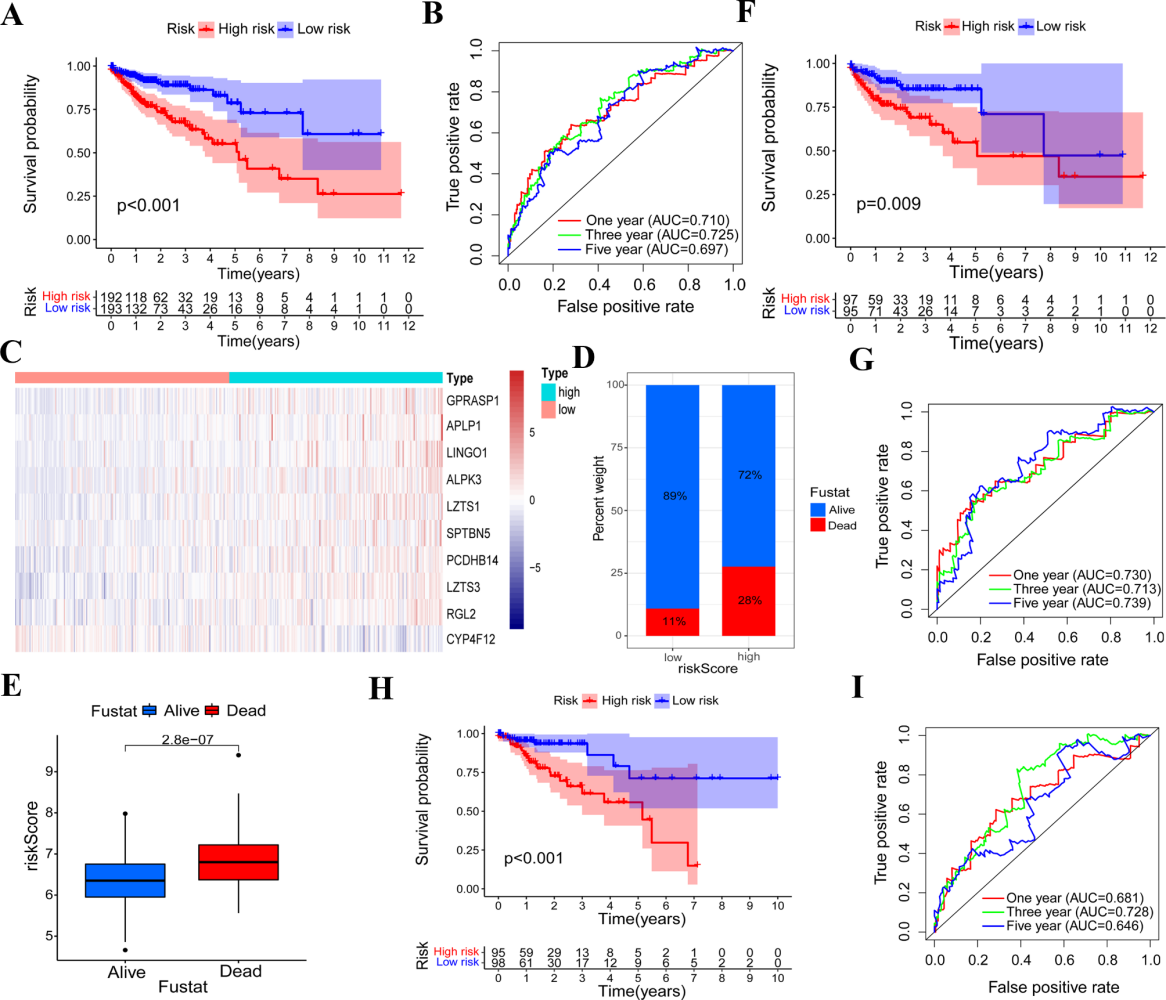
Prognostic function of evaluation and inner validation. **(A)** Survival difference between low-risk and high-risk patients, ***P < 0.001. **(B)** Prediction accuracy of the risk signature. **(C)** Expression difference of ten genes between low-risk group and high-risk group. **(D)** Survival status difference between low-risk and high-risk patients. **(E)** Risk score value difference between alive and dead patients, ***P < 0.001. **(F** and **G)** Survival difference and its corresponding AUC value in inner validation set 1, **P < 0.01. **(H** and **I)** Survival difference and its corresponding AUC value in inner validation set 2, ***P < 0.001

According to the median value of risk score, patients of TCGA cohort were clustered into a low-risk group and a high-risk group. Survival analysis showed a better prognosis in the low-risk group than that of high-risk group (Fig. 2A). The area under curve (AUC) value of the receiver operating characteristic (ROC) curve indicated a good efficiency of the risk signature (Fig. 2B). Ten TMB-related genes in the signature exhibited obviously differential expression patterns between the low-risk group and the high-risk group (Fig. 2C). A bar plot was used to visualize the percent weight of the survival status of the COAD patients. We observed that there were more deaths in the high-risk group (Fig. 2D). In addition, we found that dead patients have a relatively higher risk score than alive patients (Fig. 2E). These results suggested that the TMB-related genes’ signature could be used to predict the prognosis of patients with COAD.

Given that there are indeed several statistical methods to shrink variables [11], such as least absolute shrinkage and selection operator (LASSO), elastic net regularization (elastic net) and network-regularized high-dimensional Cox-regression (Net). To elucidate that the using of LASSO is proper in our analysis, we conducted another method (Cox-regression) to shrink variable. We obtained a new signature with six genes. This signature also exhibited an undeniable role in predicting patients’ survival (Supplementary Fig. 2A-C) in training set. However, we found that the performance of this signature in testing set (Supplementary Fig. 2D-F, with *P* = 0.035. AUC at 1, 3, 5 years = 0.559, 0.547, 0.552, respectively) is not equal with that of the signature form the LASSO analysis (Figure S1C-E, with *P* = 0.01. AUC at 1, 3, 5 years = 0.567, 0.567, 0.574, respectively). Thus, we speculate that LASSO analysis might be a better method to shrink variable in our analysis.

To further determine the prognostic function of our risk signature, we explored the performance of the signature in two inner validation sets. Results of survival analyses showed that high-risk patients have poorer OS probability than the patients with low-risk pattern in two validation sets (Fig. 2F H). The AUC values of ROC curves showed that two inner validation sets have a similar prediction accuracy exhibited in the training set (Fig. 2G and I). Apart from two inner validation sets, we also evaluated the prognostic value of the risk signature in an external GEO cohort (GSE39582). We found that low-risk patients have obviously better survival probabilities than patients with higher risk scores (Supplementary Fig. 1C and 1D). Expression patterns of these ten genes were similar with that in TCGA cohort (Supplementary Fig. 1E).

Considering that COAD is a high heterogenous cancer according to the different tumor locations [12–14]. We wonder whether our signature also functions in different locations of COAD. To verify our hypothesis, we divide COAD patients into right-side colon cancer (RCC: includes the cecum, ascending colon, and hepatic flexure) and left-side colon cancer (LCC: includes the splenic flexure, descending colon, and sigmoid colon). Then, we explored the performance of our signature. Interestingly, we found that our signature exhibited a good prognostic function both in RCC (Supplementary Fig. 3A and 3B) and LCC (Supplementary Fig. 3C and 3D), which indicated that the genes in the signature might exert crucial function both in RCC and LCC.

### Correlation between the risk score and patients’ clinical characteristics

**Fig. 3:**
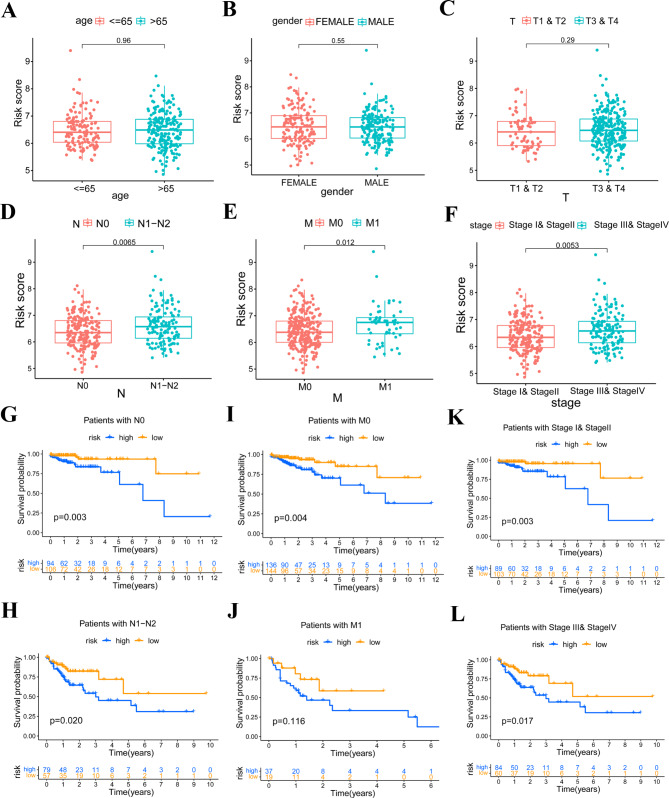
Clinical correlation and clinical characteristic specific survival analysis. **(A-F)** The correlation between risk score and patients’ clinical characteristics, *P < 0.05, **P < 0.01. **(G-L)** Survival difference between low-risk and high-risk group in patients with diverse clinical characteristics, *P < 0.05, **P < 0.01

We next evaluated the correlation between the risk score and clinical features of COAD patients. We found there were no significant correlation between risk score value and patients’ age, gender and T stage (Fig. 3A C). However, the risk score value exhibited a significantly correlation with patients’ N stage (*p* = 0.0065), M stage (*p* < 0.012), clinical stage (*p* = 0.0053) (Fig. 3D F). Patients with lymph node metastasis (N1-N2), distant metastasis (M1) and advanced clinical stage (Stage III-IV) have a higher risk score value. Considering that patients with lymph node metastasis (N1-N2), distant metastasis (M1) and advanced clinical stage (Stage III-IV) have poorer survival outcomes, we further explored the survival outcome difference in patients with different N stage, M stage and clinical stage to prove the prognostic value of the risk signature is not dependent on patients’ N stage, M stage and clinical stage. As expected, we observed that our risk signature could be used to predict patients’ survival outcome in all subgroups except in patients with M1 stage (Fig. 3G L). As for no differenced was observed in patients with M1 stage, we attributed this to the small sample size, which needed to be validated in larger samples.

### Independent prognosis analysis and nomogram construction

**Fig. 4:**
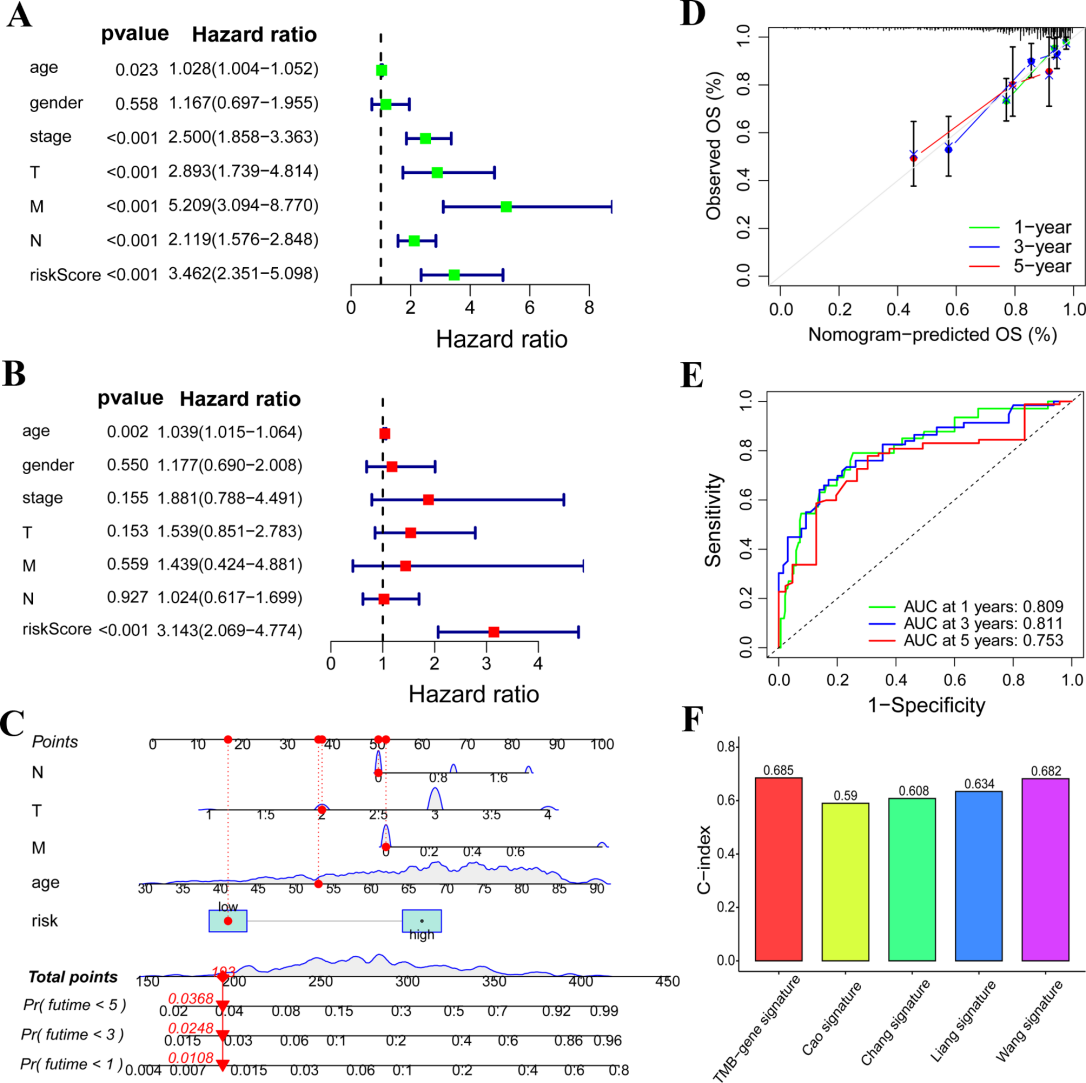
Independent prognostic function identification and survival time prediction. **(A** and **B)** Univariable Cox regression and multivariable Cox analyses were performed to screen indicator with prognostic function. **(C)** A nomogram was generated to predict patients’ overall survival time. **(D** and **E)** A calibration curve and a ROC curve were generated to evaluated the accuracy of the nomogram. **(F)** C-index was used to compared the prediction accuracy difference between TMB-gene signature and other four signature in COAD.

Our above results identified a novel risk signature to predict survival outcome of COAD patients. In clinical practice, some clinical characteristics could also be used to predict patients’ prognosis. To prove the independent prognostic value of the risk score, we determined the prognostic factors in COAD by using univariate and multivariate Cox regression analyses. The hazard ratio of clinical features and risk score was shown in Fig. 4A and B. Only risk-score exhibited a significant value in multivariate analysis (Fig. 4B). We can conclude that risk-score is the most effective independent prognostic indicator. Then, we constructed a nomogram to predict patients’ overall survival time (Fig. 4C). Calibration curves and corresponding ROC curves were plotted to assess the accuracy of the nomogram. Result of calibration curves showed that the predicted overall survival probabilities were nearly same as the observed overall survival probabilities (Fig. 4D). The AUC value of the ROC was 0.809, 0.811 and 0.753, at one year, three years and five years, respectively (Fig. 4E).

To further prove the superiority of our risk signature, we obtained four published signature and compared the prognostic value of our TMB-gene signature with other four signatures by assessing c-index difference [15–18]. Results demonstrated TMB-gene signature has the highest c-index value than other four signatures (Fig. 4F), which further proved the prognostic value of our signature.

### Validating the superiority of signature’ prognostic function and discovering of molecular functions and pathways

**Fig. 5:**
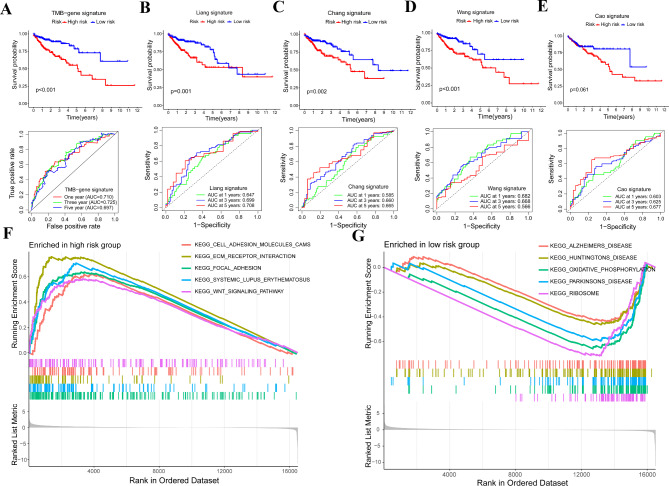
Comparing of signature’ prognostic value and discovering of molecular functions and pathways. **(A-E)** The survival difference and its’ corresponding accuracy value were characterized by using Kaplan-Meier analysis and ROC curve, **P < 0.01, ***P < 0.001. **(F** and **G)** GSEA analysis was used to obtain the enrichment of pathways in low-risk group and high-risk group (www.kegg.jp/kegg/kegg1.html)

Our above results showed that our TMB-genes’ signature might have a better prognostic value than the other four signatures. To prove our findings, we determined the survival difference of COAD patients by applying these five signatures in patients with COAD. In addition, we also constructed ROC curves to evaluate the accuracy of these five signatures. We found that four out of five signatures could significantly divide patients into two subgroups with different prognosis (Fig. 5A and D). Only Cao’s signature could not be used to predict patients’ survival in out training set (Fig. 5E). Compared with the other four signatures, the accuracy indicator (AUC value) results in our signature also indicated that our TMB-gene signature has the best prognostic performance (Fig. 5A and E).

To better understand the potential mechanism differences between the low-risk group and the high-risk group, we conducted gene set enrichment analyses (GSEA) and defined the KEGG enrichment differences between two groups. Results demonstrated that tumor progression related pathways such as ‘CELL ADHESION MOLECULES CAMS’, ‘ECM RECEPTOR INTERACTION’, ‘FOCAL ADHESION’ and ‘WNT SIGNALING PATHWAY’ were mainly enriched in high-risk group (Fig. 5F and G), which is consistent with the poor prognosis of these patients. These results suggested that poor prognosis of high-risk group patients might be associated with the tumor progression, which was potentially closely correlated with the status of the COAD microenvironment.

### Correlation between the signature and tumor dryness, microsatellite instability and drug sensitivity

**Fig. 6:**
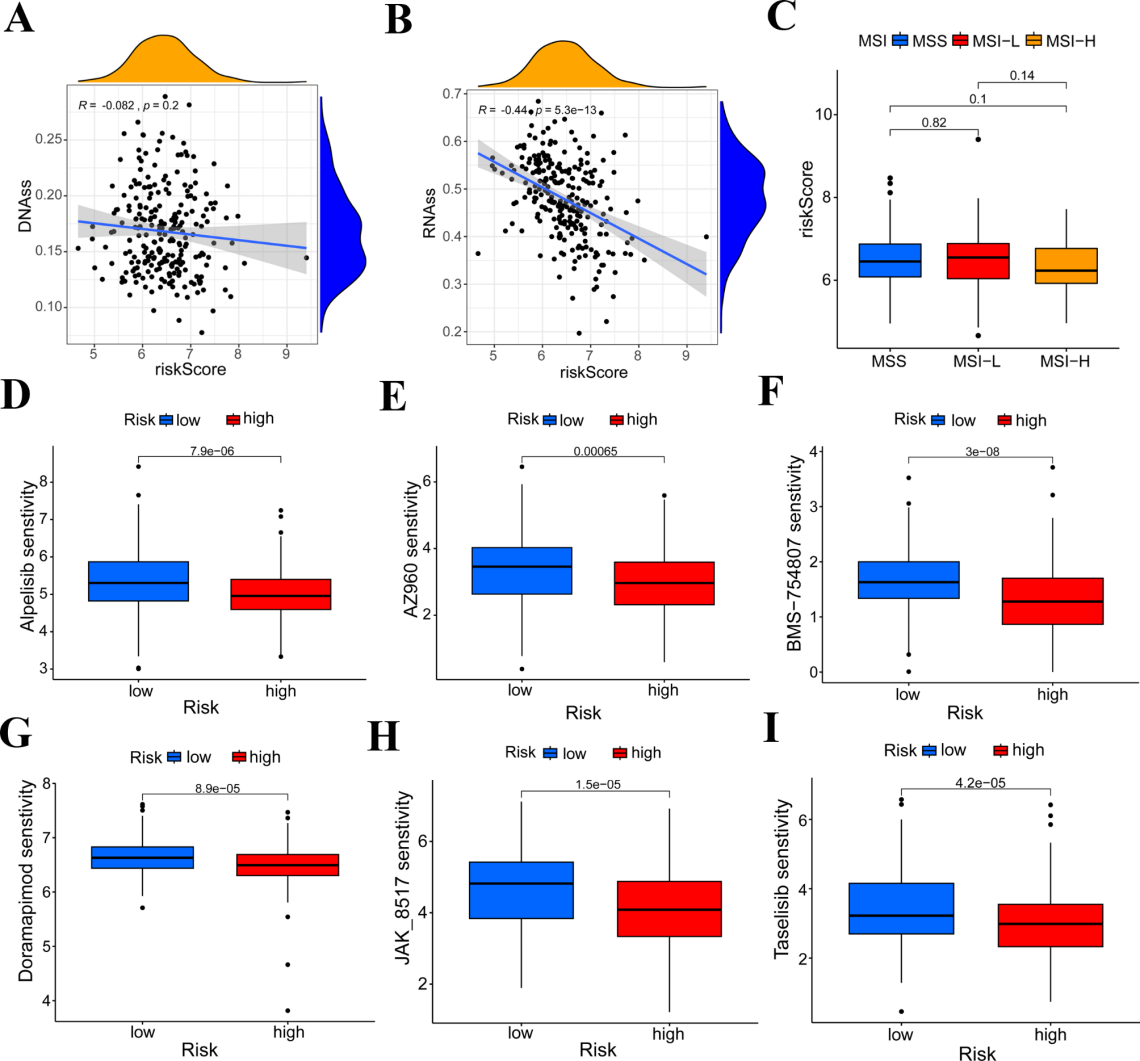
Correlation between the signature and tumor dryness, genomic instability and drug sensitivity. **(A)** The signature is nor correlated with the DNAss of COAD patients. **(B)** The risk score of the signature is negatively correlated with the RNAss of COAD patients, ***P < 0.001. **(C)** The risk score of the signature is not correlated with the MSI of COAD patients. (D-I) Patients with low risk are more sensitive to Alpelisib, AZ960, BMS-754,807, Doramapimod, JAK_8517 and Taselisib, ***P < 0.001

Apart from the prognostic function, we also explored the correlation between the signature and some characteristics of tumor including tumor dryness, microsatellite instability and drug sensitivity. We found that the risk score not correlated with the DNAss score of COAD patients (Fig. 6A). However, the risk score is negatively correlated with the RNAss score of COAD patients (Fig. 6B). We also observed that the risk score is not correlated with the microsatellite instability (MSI) status of COAD patients (Fig. 6C). Results of drug sensitivity showed that patients with low risk are more sensitive to PI3K inhibitor Alpelisib, JAK inhibitor AZ960, IGF-1R inhibitor BMS-754,807, MAPK inhibitor Doramapimod, JAK inhibitor JAK_8517 and PI3KCA inhibotor Taselisib (Fig. 6D-I). These results proved that the signature might have potential function in predicting the tumor dryness and drug sensitivity of COAD patients.

### Association of the signature with the proportion of TICs

**Fig. 7:**
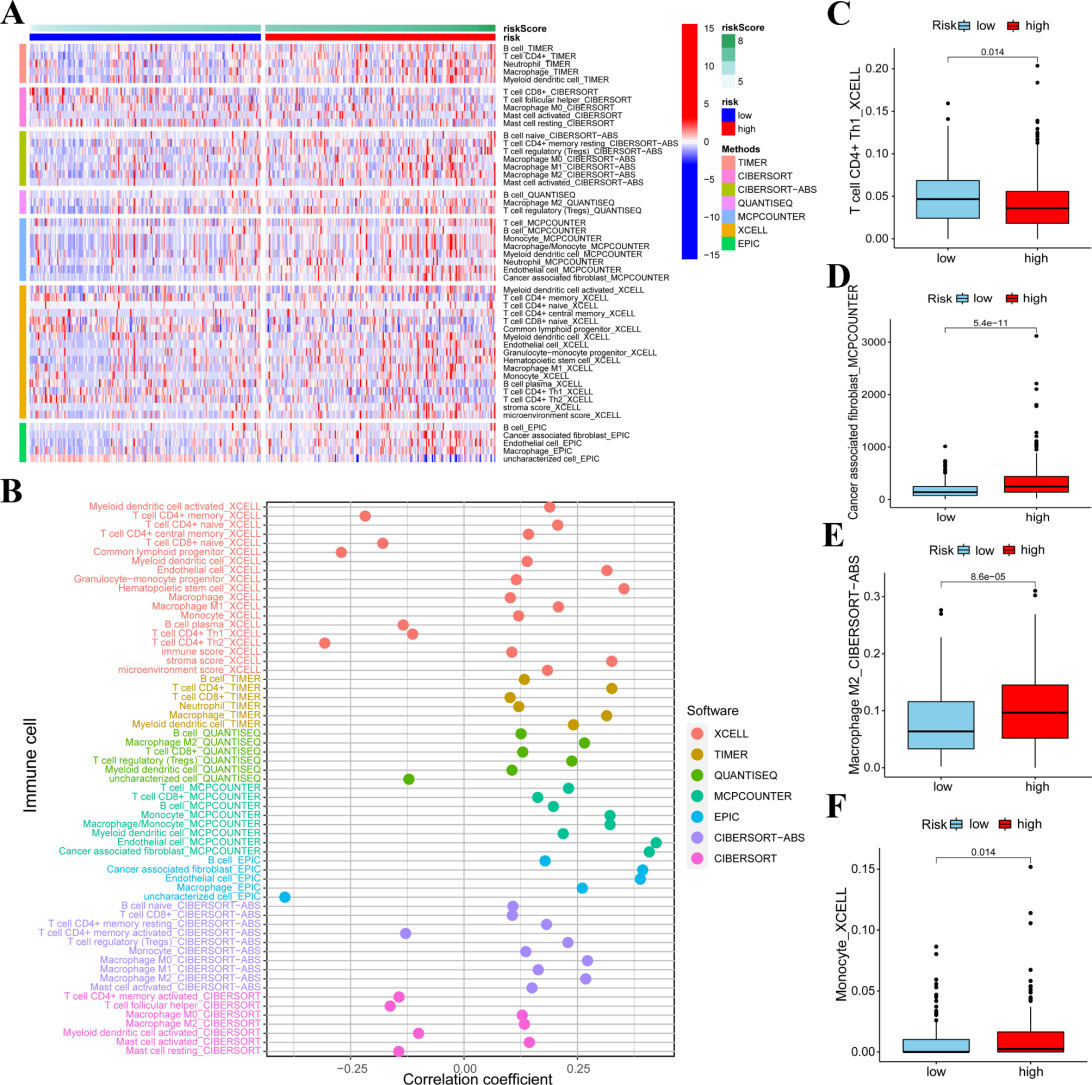
Immune cell infiltration difference characterized by the risk signature. **(A)** Immune cell infiltration was assessed and visualized with a heatmap. **(B)** The correlation between risk score value and immune cells infiltration status. **(C-F)** The infiltration difference of ‘Tell CD4 + Th1’, ‘cancer associated fibroblast’ ‘macrophage M1’ and ‘Monocyte’ between two groups were visualized, *P < 0.05, ***P < 0.001

To further explore the correlation of the risk signature with the immune microenvironment, we acquired the immune cell infiltration data of COAD patients from TIMER2.0 (https://timer.comp-genomics.org). After obtained the immune cell infiltration data, we next compared the infiltration differences of immune cells by using the methods of ‘TIMER’, ‘CIBERSORT’, ‘CIBERSORT-ABS’, ‘QUANTISEQ’, ‘MCPCOUNTER’, ‘XCELL’ and ‘EPIC’. Interestingly, we found that the infiltration of ‘macrophage’, ‘macrophage M2’, ‘Monocyte’, ‘cancer associated fibroblast’ and ‘Mast cell activated’ was positively correlated with the risk score value in the results of most algorithms. The infiltration of ‘T cell CD4 + activated’, ‘T cell CD8 + activated’ and ‘Tell CD4 + Th1’ were negatively correlated with the value of risk score (Fig. 7A and B). The infiltration difference of tumor progression related immune cells (including ‘Tell CD4 + Th1’, ‘cancer associated fibroblast’ ‘macrophage M2’ and ‘Monocyte’) between two groups were shown in Fig. 7C-6 F.

### Protein expression validation of the genes in our signature

**Fig. 8:**
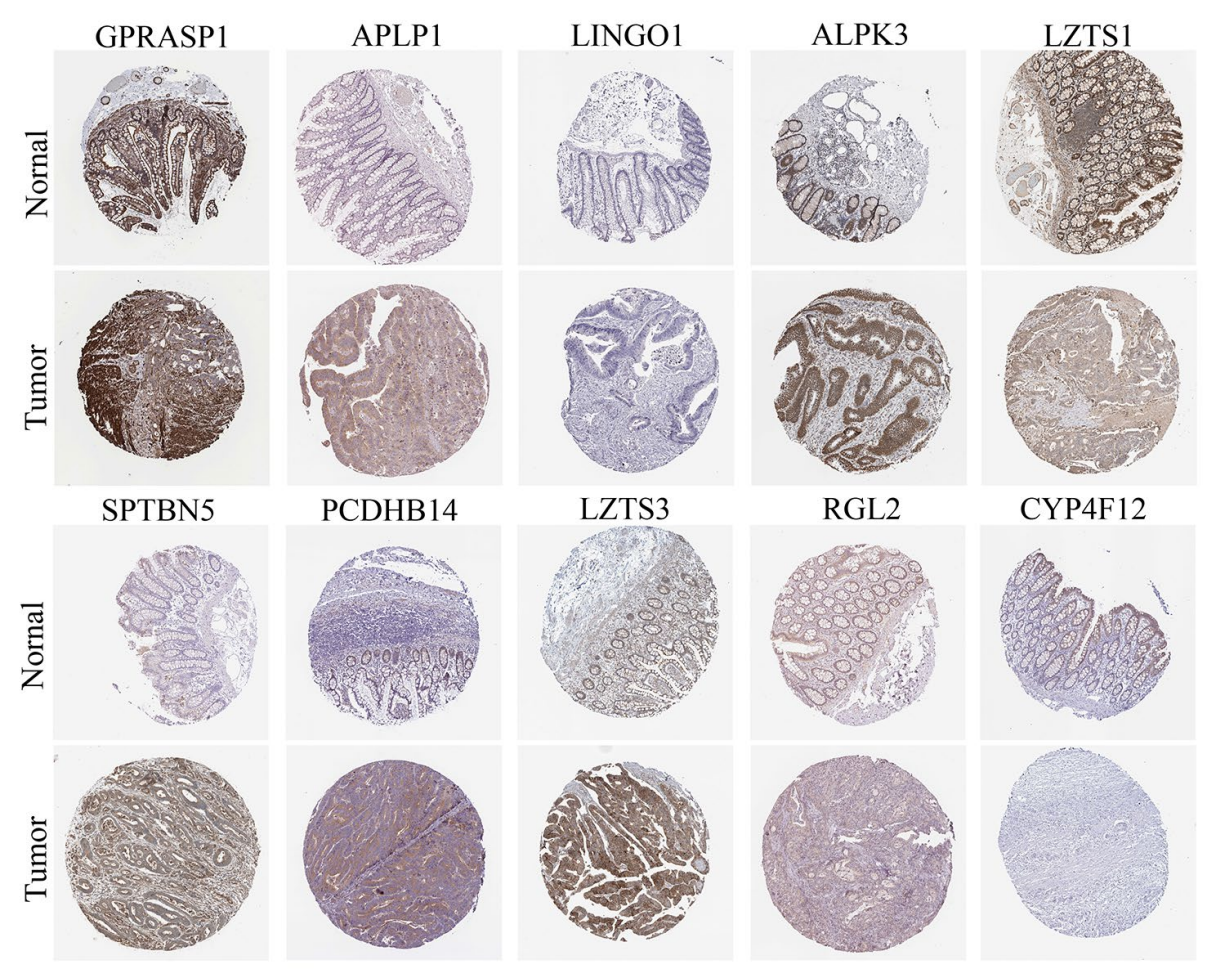
Protein expression validation of ten genes in clinical samples from online website dataset

Our signature identified that ten genes were closed corelated with patients’ survival. To further validate the correlation between these genes and COAD, we acquired the protein expression of these ten genes from https://www.proteinatlas.org/. We found that most genes with hazard ratio > 1 (GPRASP1, APLP1, ALPK3, SPTBN5, PCDHB14, LZTS3 and RGL2) have a higher expression in tumor tissues than normal tissues except LINGO1 and LZTS1 (Fig. 8). There was no difference observed in LINGO1 between tumor tissues and normal tissues. LZTS1 exhibited a lower protein level in tumor tissues than normal tissues. In addition, we found that the gene with hazard ratio < 1 (CYP4F12) show a lower protein level in tumor tissues. Thus, we speculated that GPRASP1, APLP1, ALPK3, SPTBN5, PCDHB14, LZTS3, RGL2 and CYP4F12 might exert a more crucial role in COAD progression.

## Discussion

TMB was defined as the number of non-synonymous somatic coding errors per megabase in cancer cells [19]. Mutations of driver genes might be associated with the occurrence and malignant degree of tumors. COAD is a tumor with high heterogeneity, which resulted from a series of genetic mutation in tumor cells [3, 4]. Owing to the high heterogeneity of most tumors, there has been an increasing number of studies focus on the roles of the tumor mutation (TMB) in tumors currently. Among these studies, most studies focus on the role of TMB in prognostic prediction and immune landscape. Circulating tumor DNA TMB was identified as a viable indicator to predict immunotherapy efficiency in non-small cell lung cancer [20]. The level of TMB was utilized to predict clinical outcomes of melanoma [21]. TMB was also reported to exert crucial functions in digestive tract cancer. Fu found that TMB plays a significant role in the prognosis of GC patients [22]. In COAD, TMB was identified to be associated with the drug sensitivity and immune infiltration [23, 24]. However, the detail role of TMB-related genes in COAD has not been previously discussed.

In the recent years, various analytical approaches such as Cox regression analysis, PPI analysis and other bioinformatics methods were used to identify the genes associated with the prognosis of cancer patients [25–28]. To identify a TMB-related genes’ signature of COAD, we conducted a various of bioinformatics analyses and identified a novel risk signature using ten TMB-related genes (GPRASP1, APLP1, ALPK3, SPTBN5, PCDHB14, LZTS3, RGL2, LINGO1, LZTS1 and CYP4F12). Among these genes, GPRASP1 was correlated to the prognosis of glioblastoma patients [29]. ALPK3 was identified to be associated with the metastasis of osteosarcoma patients [30]. PCDHB14 was revealed to be differentially expressed between normal tissue and tumor tissue of colon [31]. However, the function of PCDHB14 remains poorly understood. LZTS3 was proved to be a tumor suppressor in lung cancer [32]. A recent study reported that RGL2 could drive the metastatic progression of colorectal cancer via preventing the degradation of β-Catenin and KRAS [33]. LINGO1 was identified as prognostic predicting indicator in primary glioblastoma [34]. LZTS1 has been previous reported to suppress colorectal cancer proliferation and metastasis [35, 36]. However, LZTS1 was identified as a poor prognostic indicator of COAD. We attribute this difference to the reason that we only include COAD patients, other studies included patients with COAD and rectum cancer. The biological functions of COAD and rectal cancer might be different. Genetic variants in CYP4F12 were associated with glioma susceptibility [37]. In addition, CYP4F12 was proved as an indicator in the prognosis and immune infiltration of cervical cancer [38]. These evidences further proved that the genes in our signature exert crucial in tumor progression. Then, we explored the prognostic predicting function of the signature. Results demonstrated that our signature exerts an undeniable role in predicting the prognosis of COAD patients. Results of two inner validations and an external validation also proved that our signature functions in COAD patients. Moreover, we found that out signature have good prognostic function both in RCC and LCC. These results indicated that the risk signature might have the application value in all COAD patients. Subsequently, we screened indicators with prognostic function and constructed a nomogram to predict overall survival outcomes of COAD patients. To prove the superiority of our signature, we acquired four published signatures in COAD and compared the prediction accuracy. Results suggested that our TMB-gene signature has the best prognostic value.

To better understanding the underlying mechanism differences between the low-risk group and the high-risk group, we conducted GSEA analyses and found ‘CELL ADHESION MOLECULES CAMS’, ‘ECM RECEPTOR INTERACTION’, ‘FOCAL ADHESION’ and ‘WNT SIGNALING PATHWAY’ were enriched in high-risk group. These pathways were reported to be associated with the progression of multiple tumors [39–43], which further proved that tumors in high-risk patients are more invasive. A recent study found that COAD patients with the enrichment of ‘CELL ADHESION MOLECULES CAMS’ have a poorer survival outcome [44]. As for the ‘ECM RECEPTOR INTERACTION’ regulatory network, it was revealed to exert crucial function in COAD development and metastasis [45]. WNT SIGNALING PATHWAY was the classical tumor related pathway associated with gastrointestinal tumors. Multiple tumor related genes could activate WNT signaling to affect tumor progression [46, 47]. Drugs targeting WNT signaling were proved to have potential clinical application value in COAD patients [48]. Apart from the prognostic function, we found that the signature is correlated with the tumor dryness and drug sensitivity of COAD patients. The risk score is negatively correlated with the RNAss score of COAD patients. Usually, patients with higher dryness score have a more invasive tumor. However, our patients with high risk have a lower RNAss score. The application of our signature in tumor dryness requires more validation. From the results of drug sensitivity, we found that patients with low risk are more sensitive to PI3K inhibitor, JAK inhibitor, IGF-1R inhibitor, MAPK inhibitor, JAK inhibitor and PI3KCA inhibitor, which indicated that low risk COAD patients might benefit from these drugs.

We also analyzed the function of the signature in immune microenvironment and found that our signature is correlated with the immune cell infiltration of COAD. Our results showed that high-risk patients have a more infiltration of ‘macrophage’, ‘macrophage M2’, ‘Monocyte’, ‘cancer associated fibroblast’ and ‘Mast cell activated’ in most algorithms. The infiltration of ‘macrophage’, ‘macrophage M2’, ‘Monocyte’, ‘cancer associated fibroblast’ and ‘Mast cell activated’ is associated with cancer progression, including COAD [49–52]. More specifically, Wang reported that macrophage and monocyte expressed with Tim3 might exert crucial functions in tumor progression [49]. Macrophage M2 polarization was reported to promotes colorectal cancer cells growth [53]. Cancer associated fibroblast was proved to promote colon cancer angiogenesis via increasing WNT2 [54]. In the results of GSEA also proved that WNT SIGNALING PATHWAY was activated in the high-risk group. We speculated that cancer associated fibroblast might be associated with of the activating of WNT SIGNALING PATHWAY in the high-risk group. We also observed that the infiltration of ‘T cell CD4 + activated’, ‘T cell CD8 + activated’ and ‘Tell CD4 + Th1’ were higher in the low-risk group. Infiltration of T cell CD4 + activated is helpful for maintaining the viability of NK cells [55, 56], which exert tumor inhibition functions. The infiltration of CD8 + activated T cell and CD4 + Th1 cell was also reported to exert tumor inhibition functions in tumors [57]. Results of tumor immune cell infiltration further supported that high-risk patients have poorer survival outcomes than the low-risk group. Additionally, we evaluated the protein level of these ten genes in our signature by using online database. The expression of GPRASP1, APLP1, ALPK3, SPTBN5, PCDHB14, LZTS3, RGL2 and CYP4F12 these eight genes are consistent with the hazard ratio observed in our signature. Protein level of tumor promoting genes (GPRASP1, APLP1, ALPK3, SPTBN5, PCDHB14, LZTS3, RGL2) were higher in tumor tissues. Protein level of CYP4F12 was higher in normal tissues. Thus, we speculated that these eight genes might play a more key role in the signature. Further studies on are required to explore the detail function of these genes.

In conclusion, we identified a novel TMB-related signature in COAD. Our risk signature could be utilized to predict the prognosis and immune cell infiltration of COAD patients. Our findings might provide evidences for the clinical judgement of prognosis and development of new treatment strategies.

## Materials and methods

### Data source and acquiring of TMB-related genes

The tumor mutation burden data was downloaded from The Cancer Genome Atlas (TCGA). Perl scripts and R software were utilized to extract tumor mutation data from patients’ data. The detail mutation information of each gene was shown in Supplementary Table 4. Genes with mutation frequency more than in ten samples were identified as TMB-related genes (2741 TMB-related genes). Then, we obtained the RNA-sequence data from TCGA (including 39 normal samples and 404 tumor samples) and conducted expression analysis to identified differently expressed TMB-related genes between normal tissues and tumor tissues with a filter condition of *P* < 0.05, log_2_|FC|>1, which resulted in 410 differentially expressed genes (including 208 upregulated genes and 202 downregulated genes).

### Construction and validation of the signature

The total TCGA cohort was used as the training set and utilized to construct the risk signature. For the construction of the risk signature, 410 differentially expressed genes were first subjected to the univariate Cox analysis with the filter of *P* value < 0.01 (Tumor patients with unknown survival information were excluded), which result in ten genes with prognostic function. Subsequently, we conducted the LASSO Cox regression analysis on these ten genes to avoid co-linear influences and acquire regression coefficients value. The coefficients value of these ten genes were shown in Supplementary Table 5. Based on the coefficients value, the risk score of each patient was calculated as the following formula.$Risk score=\sum n coefi*\chi i$

where *xi* and *coefi* represent the expression value of each gene and its corresponding coefficient value, respectively. Multi-Cox analyses was conducted in the TCGA cohort to acquire a new signature, and the function of multi-Cox signature was tested in TCGA cohort and GEO cohort to prove that the TMN-related signature from LASSO analyses has a better prognostic predicting function.

According to the median value of the risk score, all 395 COAD patients were divided into a low-risk group and a high-risk group. Kaplan-Meier analysis was generated to compare the overall survival differences between the low-risk group and the high-risk group by using R package of ‘survival’ and ‘survminer’. A time dependent receiver operating characteristic (ROC) curve was generated to evaluate the predicting accuracy of the signature with the R package of ‘timeROC’. Expression pattern differences of these ten gene between the low-risk and the high-risk group was shown with a heat map via R package of ‘pheatmap’. The survival status of COAD patients with different risk scores were visualized with a bar plot via R package of ‘plyr’, ‘ggplot2’ and ‘ggpubr’.

As for the validation of the risk signature, patients in TCGA cohort were randomly divided into a validation set 1 and a validation set 2 at a ratio of 1:1. These two sets were used as inner validation set. COAD patients in TCGA cohort were also divided into right-side colon cancer (RCC: includes the cecum, ascending colon, and hepatic flexure) and left-side colon cancer (LCC: includes the splenic flexure, descending colon, and sigmoid colon). The function of the signature in RCC and LCC were also tested by using the method used in the training set. In addition, we also acquired data from the National Center for Biotechnology Information (GSE39582). GSE39582 cohort was used as an external validation set. Kaplan Meier curve and its corresponding accuracy value were calculated by using the method used in the training set.

### Clinical correlation of the signature

The clinical data of COAD patients was obtained from The Cancer Genome Atlas (TCGA). Clinical information of all patients were extracted via Perl script. Patients with unknown formation of any clinical characteristics were excluded. Then, the correlation between the risk score and clinical characteristics of COAD patients was calculated via ‘ggpubr’ package. The overall survival differences of patient with diverse clinical characteristics between the low-risk group and high-risk group were generated via ‘survival’ and ‘survminer’ packages.

### Survival prediction based on the signature

Univariate Cox analysis and multivariable Cox analysis were carried out to screen the prognostic indicators including the age, gender, tumor stage, and node-metastasis (N, M) stage, clinical stage and risk score in COAD. Subsequently, we generated a nomogram to predict the survival time of COAD patients. The accuracy of the nomogram was evaluated with a calibration curve and ROC curve. R package of ‘survival’, ‘survminer’, ‘timeROC’, ‘rms’ and ‘regplot’ were used in above analyses. We also acquired four COAD prognostic signatures and compared the prediction accuracy between our TMB gene signature and these four signatures.

### Gene set enrichment analysis (GSEA) based on the signature

Based on the gene expression patterns of the low-risk group and high-risk group. We analyzed the differences in the KEGG enrichment of two groups. The detected KEGG pathway were annotated using the KEGG (www.kegg.jp/kegg/kegg1.html) [58–60]. Enrichment of pathways in different risk pattern COAD patients were evaluated via R package of ‘limma’, ‘org.Hs.eg.db’, ‘clusterProfiler’ and ‘enrichplot’. Five significant pathways with *P* < 0.05 were visualized.

### Tumor dryness, microsatellite instability and drug sensitivity analyses

Tumor stemness can be measured by DNA stemness score based on DNA methylation pattern (DNAss) and RNA stemness score (RNAss) based on mRNA expression. The tumor dryness data was acquired from a previous study [61]. R package of ‘limma’, ‘ggplot2’, ‘ggExtra’ and ‘ggpubr’ were used to explore the correlation between the signature and tumor dryness of COAD patients. Microsatellite instability (MSI) data was download from the https://tcia.at/ database. The correlation between MSI and risk score were assessed by using R package of ‘plyr’, ‘ggplot2’ and ‘ggpubr’. The drug sensitivity was evaluated by using R package of ‘limma’, ‘oncoPredict’ and ‘parallel’.

### Immune infiltration analysis

As for the immune cell infiltration difference between the low-risk group and the high-risk group, we downloaded an integrated TCGA immune infiltration data (including TIMER, CIBERSORT, XCELL, QUANTISEQ, MCP counter, EPIC, and CIBERSORT) form https://timer.comp-genomics.org. Then, we visualized the immune cell infiltration status via R package of ‘limma’ and ‘pheatmap’. The correlation between the proportion of infiltrating immune cells and risk score value was evaluated and visualized via packages of ‘limma’, ‘scales’, ‘ggplot2’ and ‘ggtext’. Four bar plots were generated to show the infiltration differences of immune cells which might exert crucial roles in COAD progression.

### Statistical analysis

All data in this study were generated with Perl (5.30.1) or R (version 4.2.0) software. Survival analyses were conducted using the Kaplan-Meier method. Independent prognostic indicators for OS were screened by using univariable Cox regression analysis and multivariate Cox regression. Chi-square test were used to screen the clinical characteristics correlated to risk score.

## Electronic supplementary material

Below is the link to the electronic supplementary material.

Supplementary Material 1

Supplementary Material 2

Supplementary Material 3

Supplementary Material 4

Supplementary Material 5

Supplementary Material 6

Supplementary Material 7

Supplementary Material 8

Supplementary Material 9

## Data Availability

RNA expression data and matched clinical data and TMB files were downloaded from the TCGA (https://portal.gdc.cancer.gov/). The GEO dataset in this study was acquired with the accession number of GSE39582. Immune cell infiltration data can be found in http://timer.cistrome.org.

## Abbreviations

AGC: Advanced gastric cancer
AUC: Area Under Curve
GC: Gastric cancer
ICIs: Immune checkpoint inhibitors
IHC: Immunohistochemistry
LCC: Left-side colon cancer
NCBI: National Center for Biotechnology Information
OS: Overall survival
RCC: Right-side colon cancer
ROC: Receiver operating characteristic curve
TCGA: The Cancer Genome Atlas
TME: Tumor micro-environment
