# Supplementary figures and images for: Exploring the prognostic function of TMB-related prognostic signature in patients with colon cancer
Source: BMC Med Genomics. 2023 May 26;16:116. doi: 10.1186/s12920-023-01555-2 (PMC10214595; doi:10.1186/s12920-023-01555-2)

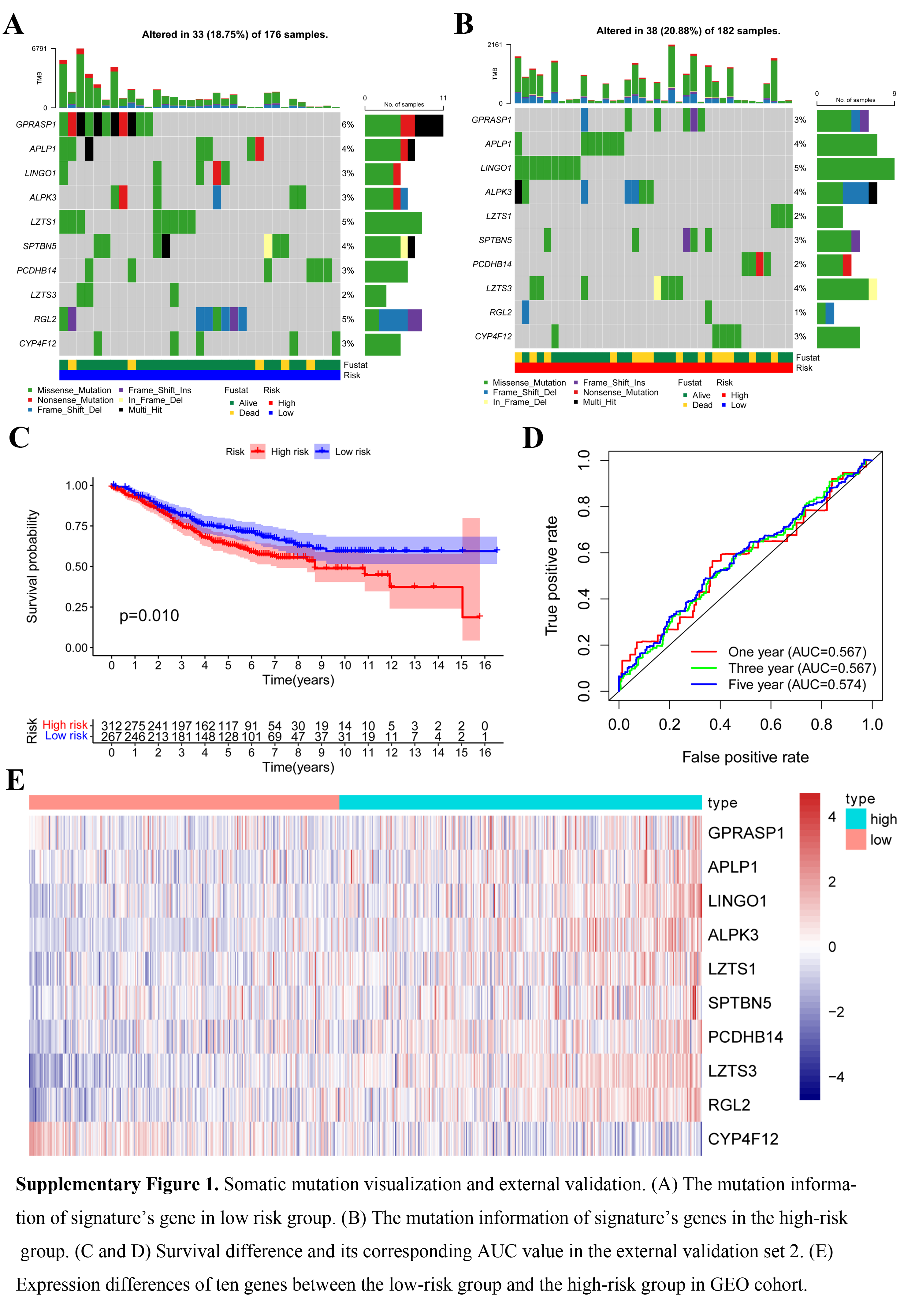

Supplement: Supplementary file 6 — Supplementary Material 6 [file 12920_2023_1555_MOESM6_ESM.tif]

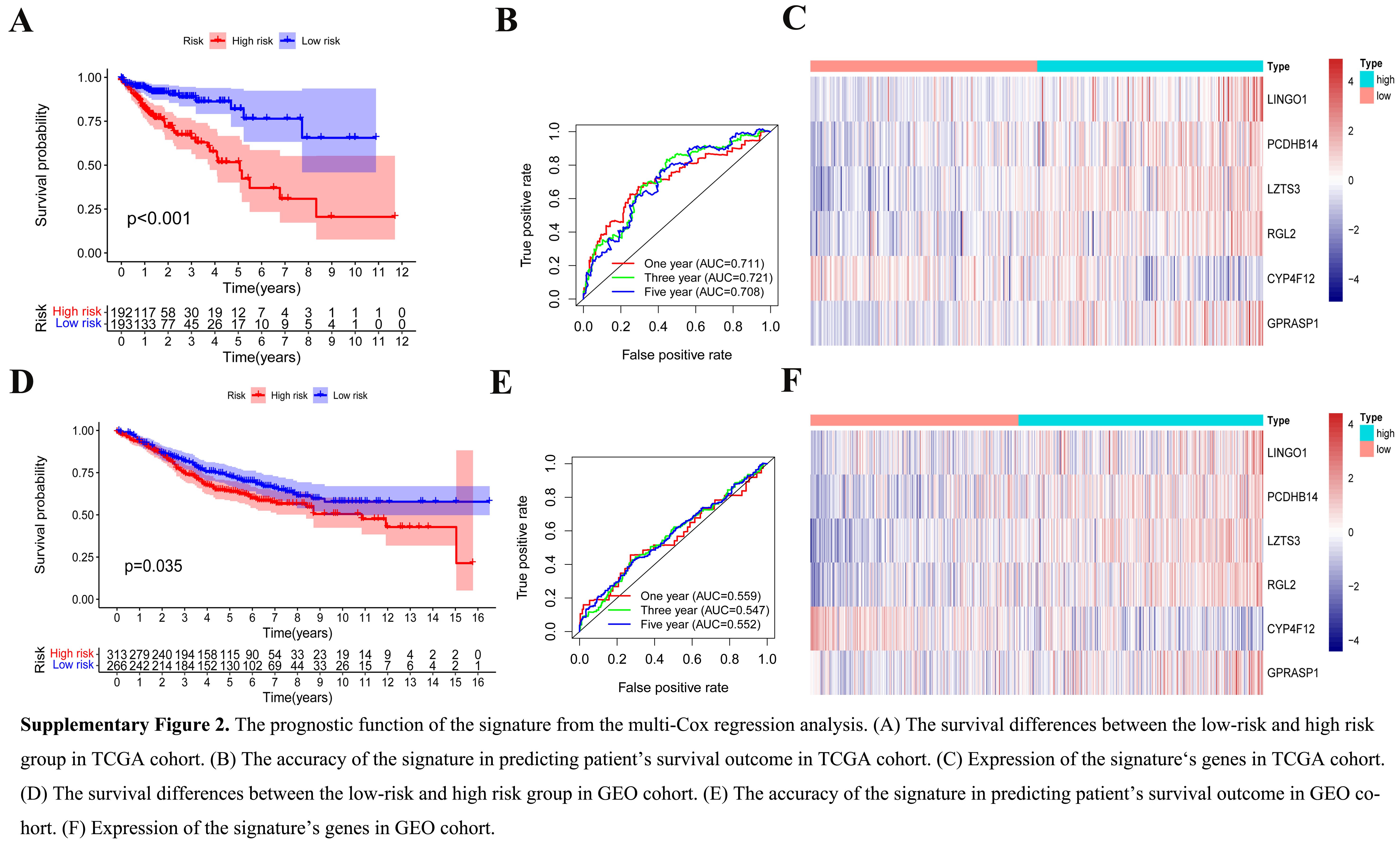

Supplement: Supplementary file 7 — Supplementary Material 7 [file 12920_2023_1555_MOESM7_ESM.tif]

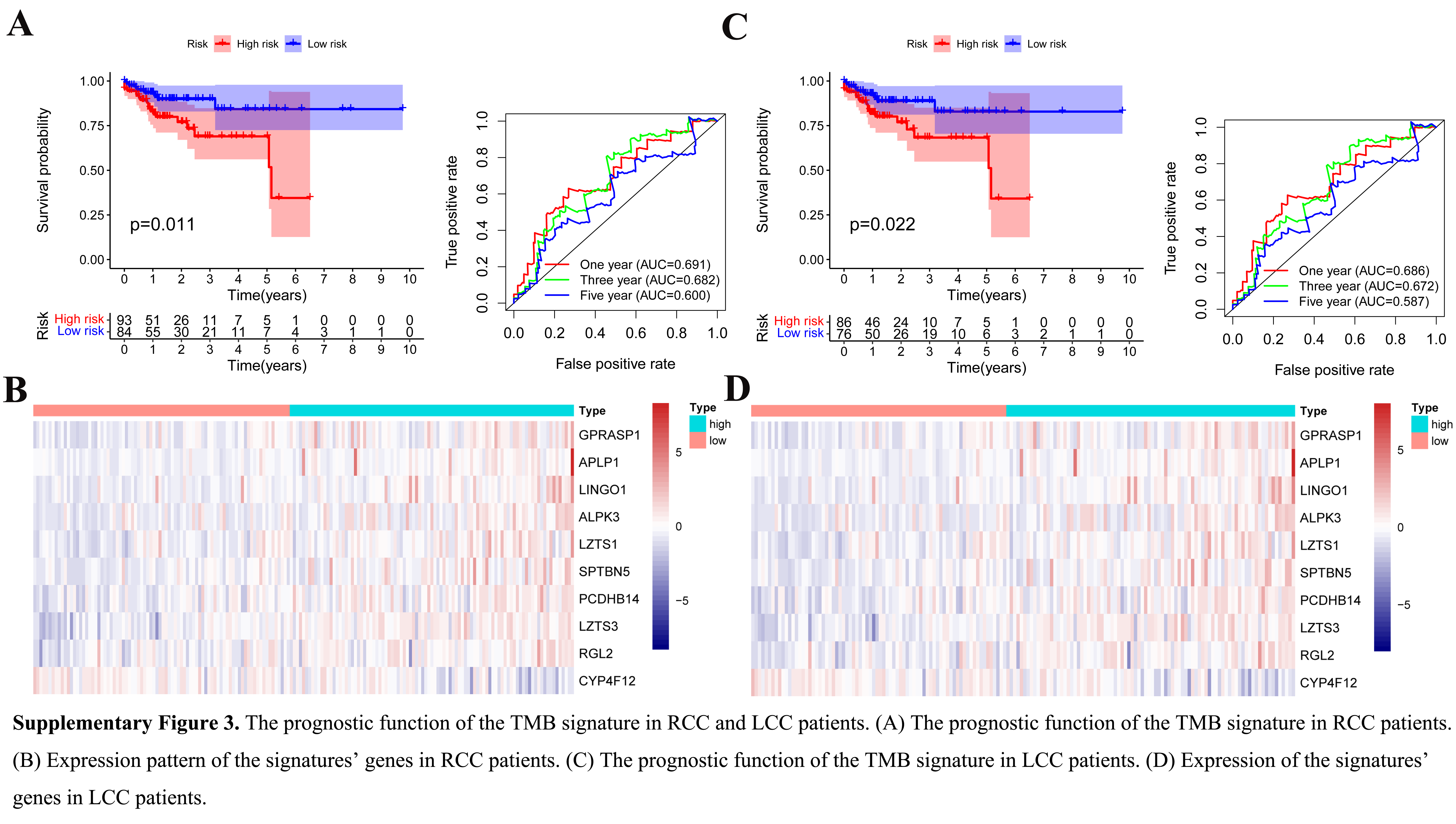

Supplement: Supplementary file 9 — Supplementary Material 9 [file 12920_2023_1555_MOESM9_ESM.tif]
