# Supplementary material for: Exploring the prognostic function of TMB-related prognostic signature in patients with colon cancer
Source: BMC Med Genomics. 2023 May 26;16:116. doi: 10.1186/s12920-023-01555-2 (PMC10214595; doi:10.1186/s12920-023-01555-2)
Supplement: Supplementary file 8 — Supplementary Material 8 [file 12920_2023_1555_MOESM8_ESM.doc]

**Supplementary Figure legends**

**Supplementary Figure 1.** Somatic mutation visualization and external validation. (A) The mutation information of signature’s gene in low risk group. (B) The mutation information of signature’s genes in the high-risk group. (C and D) Survival difference and its corresponding AUC value in the external validation set 2. (E) Expression differences of ten genes between the low-risk group and the high-risk group in GEO cohort.

**Supplementary Figure 2.** The prognostic function of the signature from the multi-Cox regression analysis. (A) The survival differences between the low-risk and the high risk group in TCGA cohort. (B) The accuracy of the signature in predicting patient’s survival outcome in TCGA cohort. (C) Expression of the signature’s genes in TCGA cohort. (D) The survival differences between the low-risk and high risk group in GEO cohort. (E) The accuracy of the signature in predicting patient’s survival outcome in GEO cohort. (F) Expression of the signatures’ genes in GEO cohort.

**Supplementary Figure 3.** The prognostic function of the TMB signature in RCC and LCC patients. (A) The prognostic function of the TMB signature in RCC patients.(B) Expression pattern of the signature’s genes in RCC patients. (C) The prognostic function of the TMB signature in LCC patients. (D) Expression of the signature’s genes in LCC patients.
